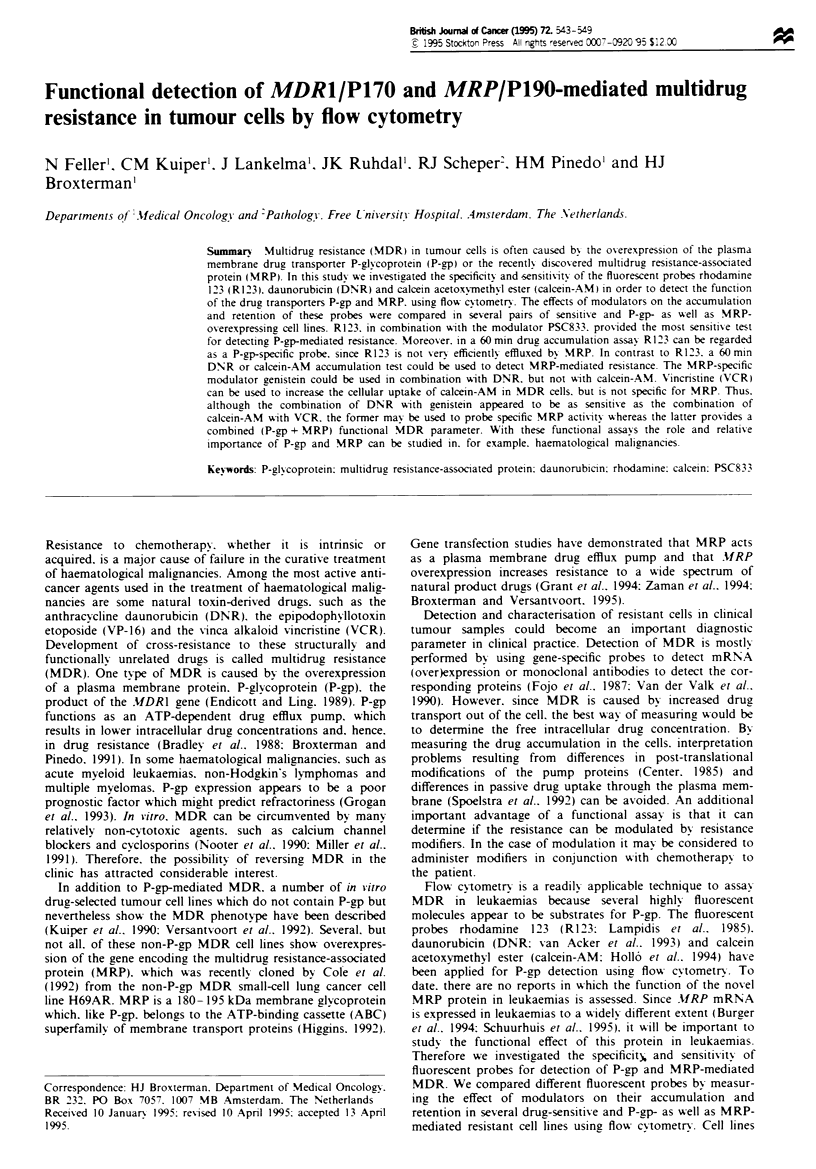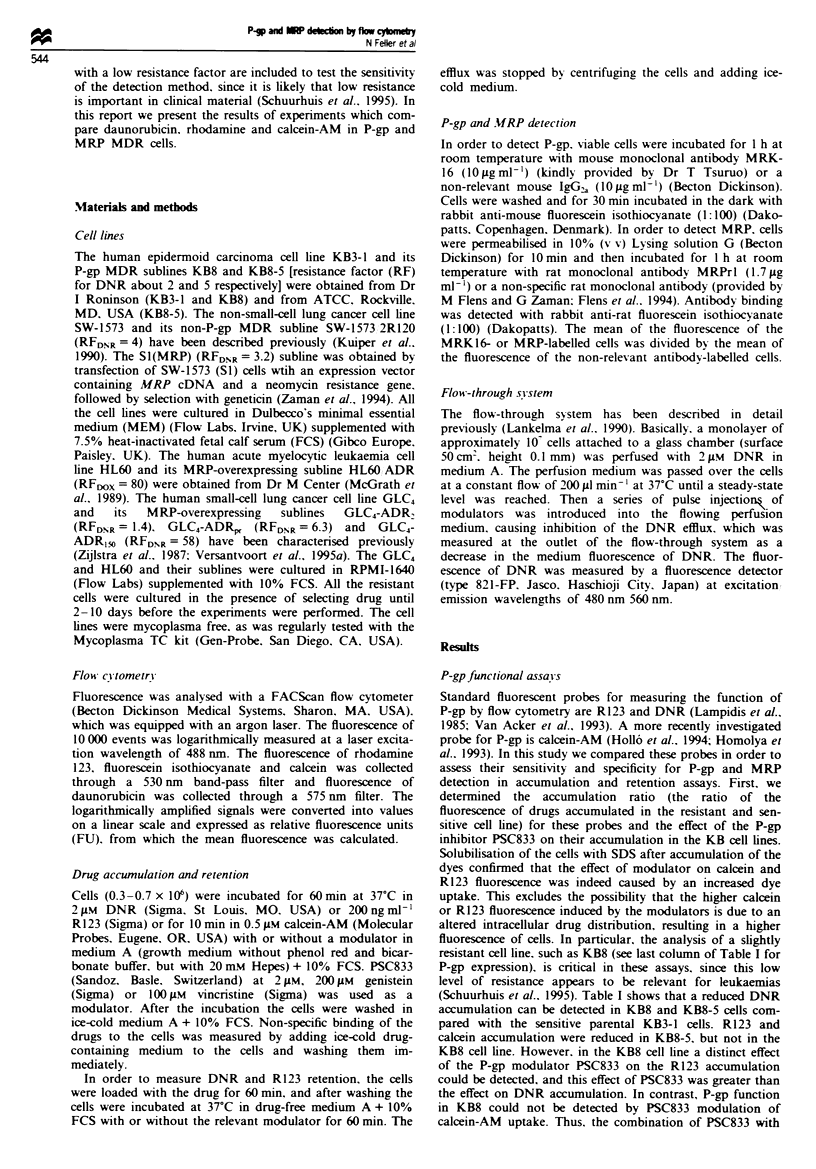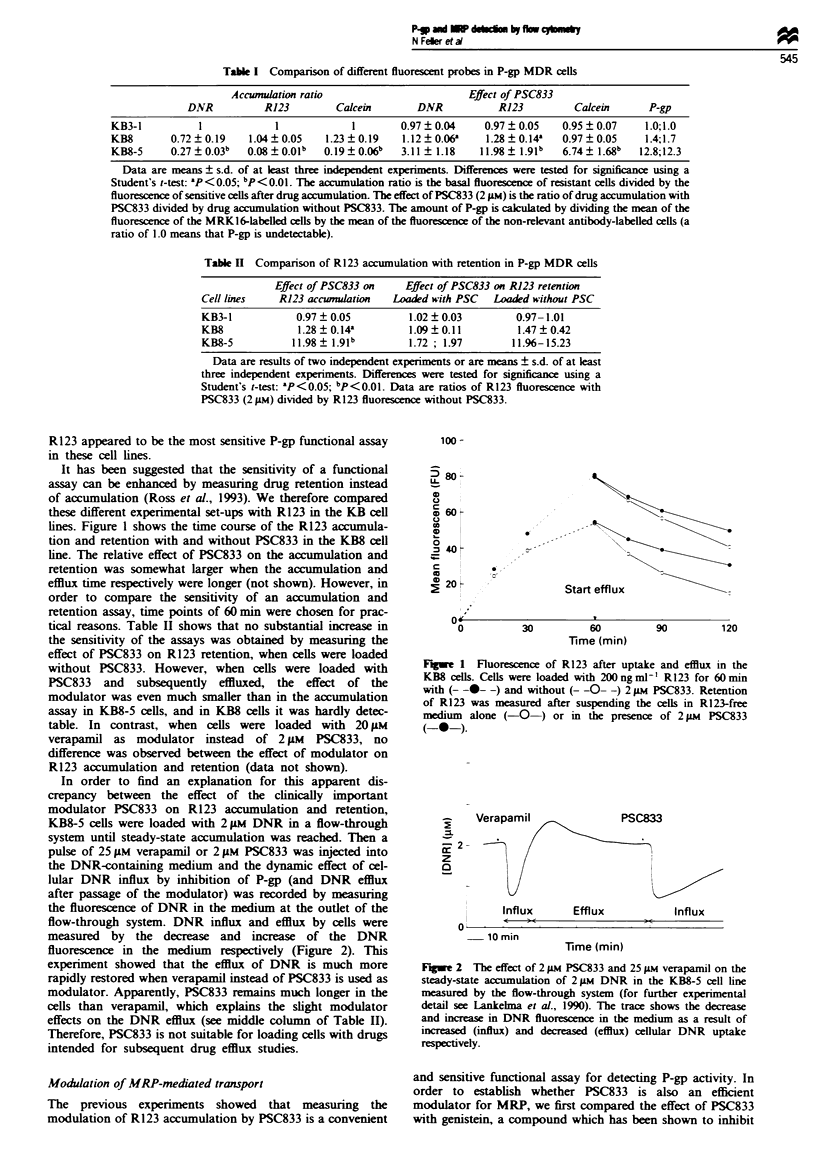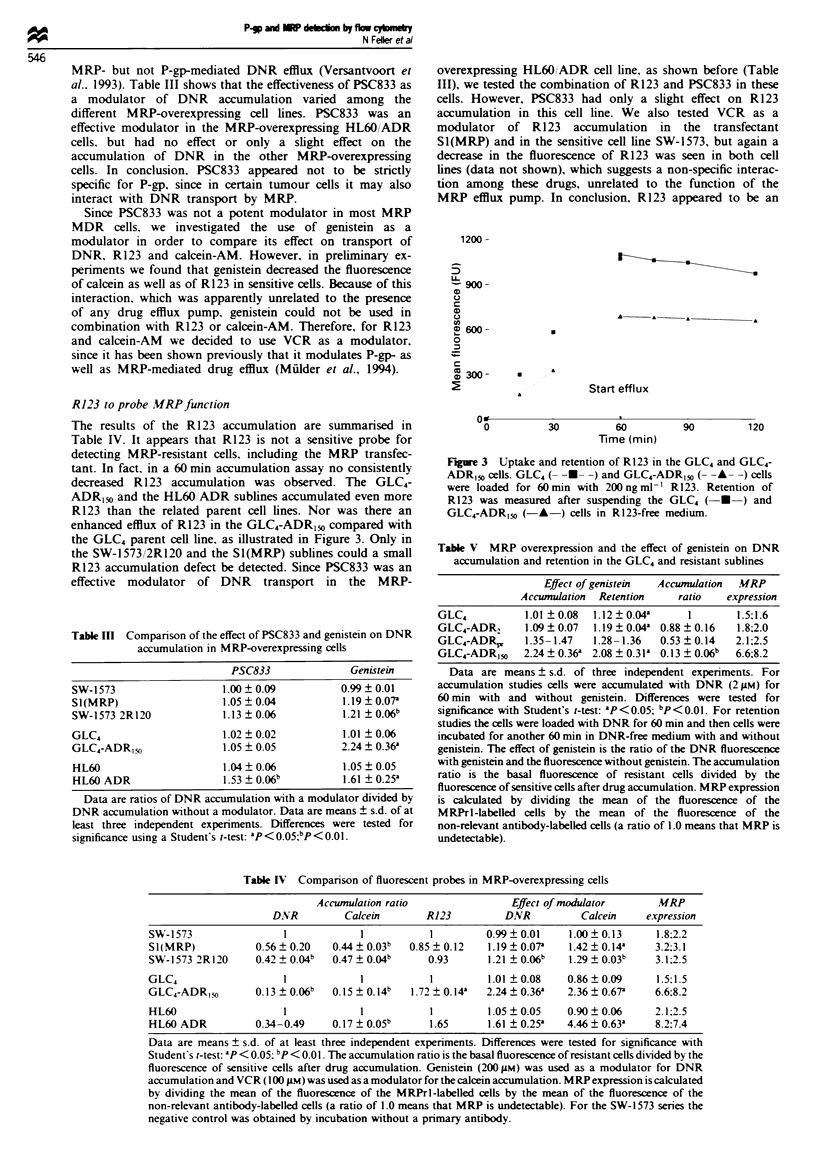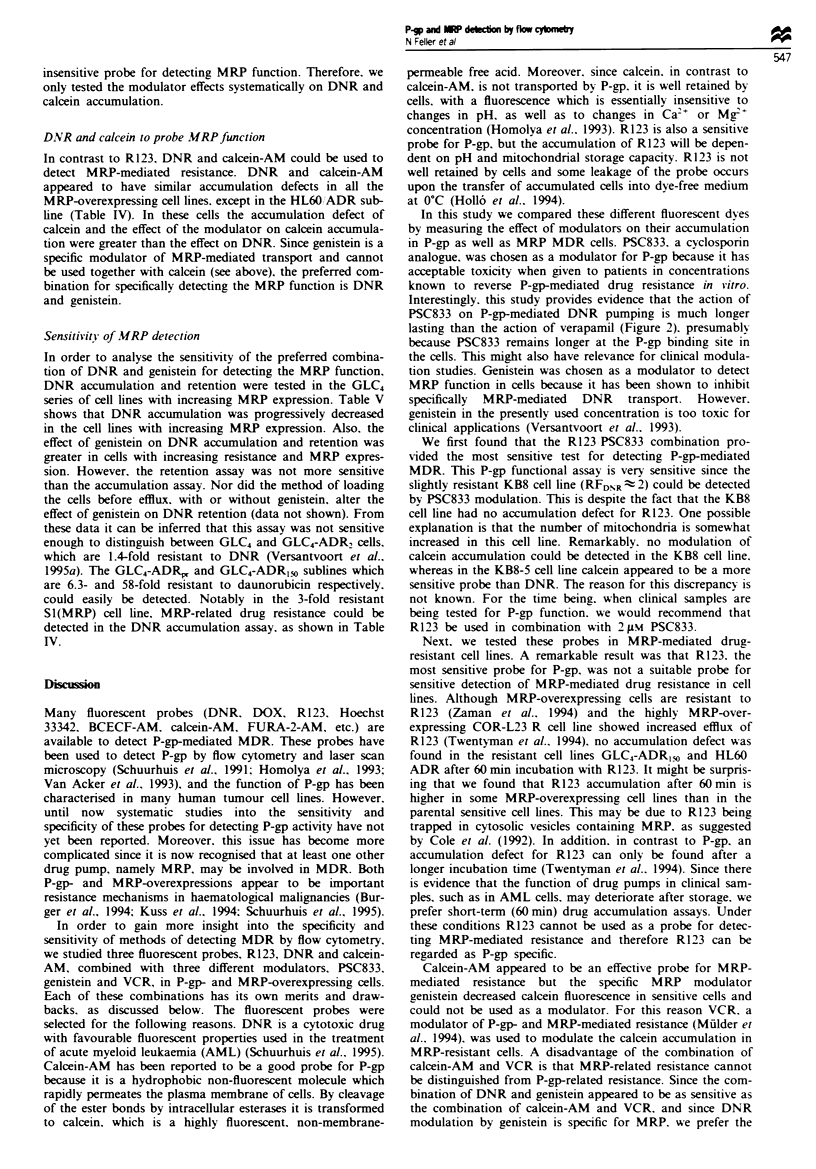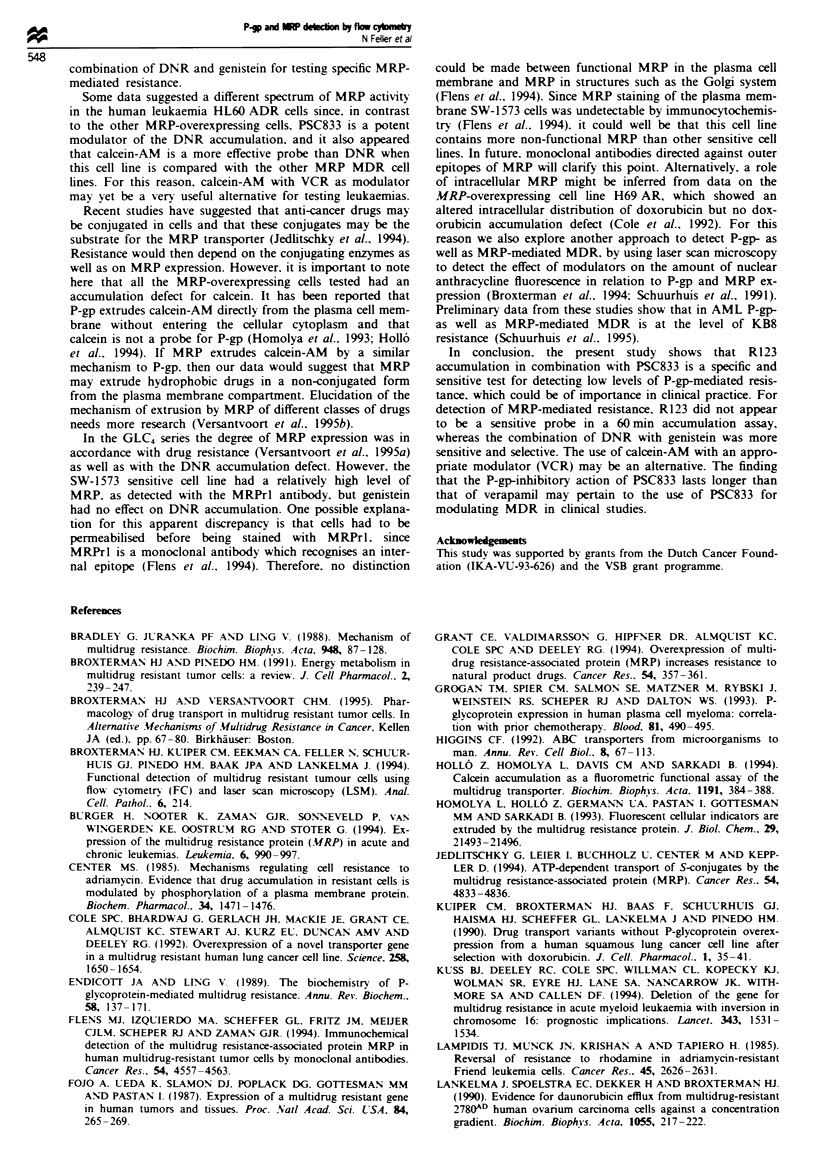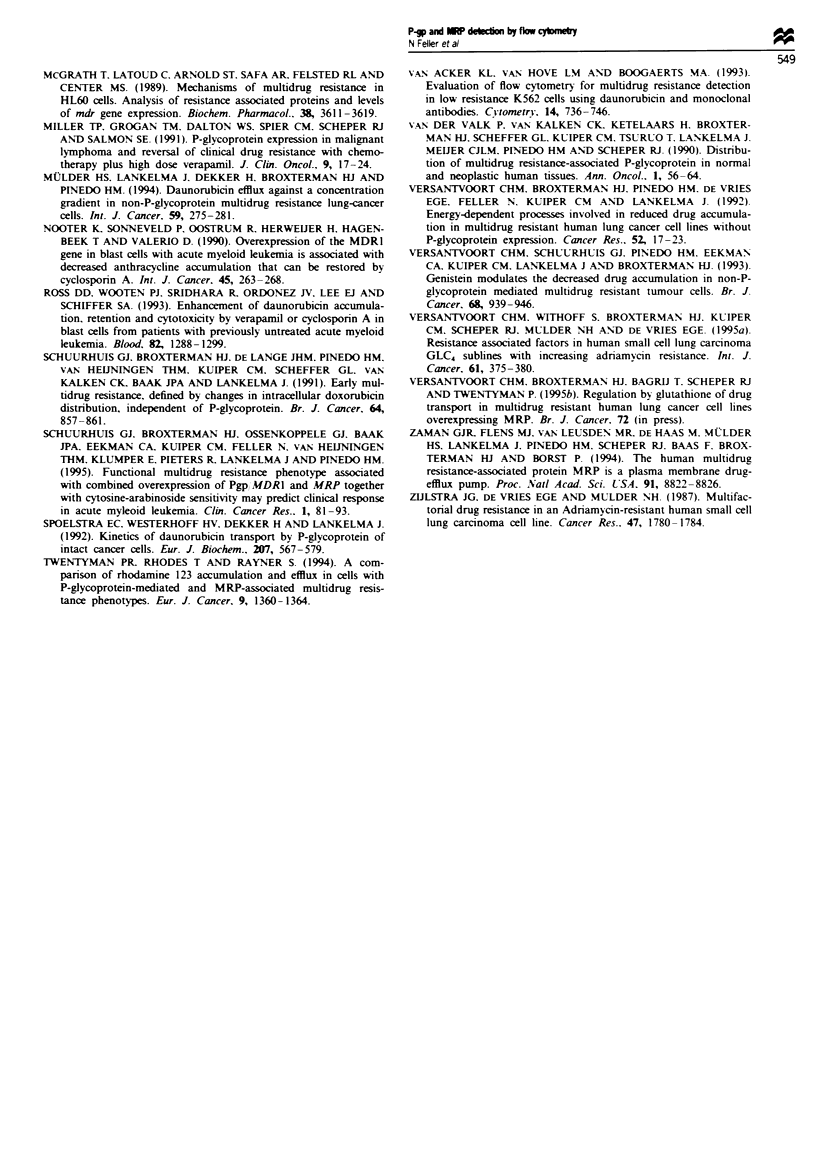# Functional detection of MDR1/P170 and MRP/P190-mediated multidrug resistance in tumour cells by flow cytometry.

**DOI:** 10.1038/bjc.1995.371

**Published:** 1995-09

**Authors:** N. Feller, C. M. Kuiper, J. Lankelma, J. K. Ruhdal, R. J. Scheper, H. M. Pinedo, H. J. Broxterman

**Affiliations:** Department of Medical Oncology, Free University Hospital, Amsterdam, The Netherlands.

## Abstract

Multidrug resistance (MDR) in tumour cells is often caused by the overexpression of the plasma membrane drug transporter P-glycoprotein (P-gp) or the recently discovered multidrug resistance-associated protein (MRP). In this study we investigated the specificity and sensitivity of the fluorescent probes rhodamine 123 (R123), daunorubicin (DNR) and calcein acetoxymethyl ester (calcein-AM) in order to detect the function of the drug transporters P-gp and MRP, using flow cytometry. The effects of modulators on the accumulation and retention of these probes were compared in several pairs of sensitive and P-gp- as well as MRP-overexpressing cell lines. R123, in combination with the modulator PSC833, provided the most sensitive test for detecting P-gp-mediated resistance. Moreover, in a 60 min drug accumulation assay R123 can be regarded as a P-gp-specific probe, since R123 is not very efficiently effluxed by MRP. In contrast to R123, a 60 min DNR or calcein-AM accumulation test could be used to detect MRP-mediated resistance. The MRP-specific modulator genistein could be used in combination with DNR, but not with calcein-AM. Vincristine (VCR) can be used to increase the cellular uptake of calcein-AM in MDR cells, but is not specific for MRP. Thus, although the combination of DNR with genistein appeared to be as sensitive as the combination of calcein-AM with VCR, the former may be used to probe specific MRP activity whereas the latter provides a combined (P-gp + MRP) functional MDR parameter. With these functional assays the role and relative importance of P-gp and MRP can be studied in, for example, haematological malignancies.


British Journal of Cancer (1995) 72, 543–549

© 1995 Stockton Press All rights reserved 0007–0920/95 $12.00

### Functional detection of *MDR*1/P170 and *MRP*/P190-mediated multidrug resistance in tumour cells by flow cytometry

N Feller[1], CM Kuiper[1], J Lankelma[1], JK Ruhdal[1], RJ Scheper[2], HM Pinedo[1] and HJ Broxterman[1]

*Departments of [1]Medical Oncology and [2]Pathology, Free University Hospital, Amsterdam, The Netherlands.*

**Summary** Multidrug resistance (MDR) in tumour cells is often caused by the overexpression of the plasma membrane drug transporter P-glycoprotein (P-gp) or the recently discovered multidrug resistance-associated protein (MRP). In this study we investigated the specificity and sensitivity of the fluorescent probes rhodamine 123 (R123), daunorubicin (DNR) and calcein acetoxymethyl ester (calcein-AM) in order to detect the function of the drug transporters P-gp and MRP, using flow cytometry. The effects of modulators on the accumulation and retention of these probes were compared in several pairs of sensitive and P-gp- as well as MRP-overexpressing cell lines. R123, in combination with the modulator PSC833, provided the most sensitive test for detecting P-gp-mediated resistance. Moreover, in a 60 min drug accumulation assay R123 can be regarded as a P-gp-specific probe, since R123 is not very efficiently effluxed by MRP. In contrast to R123, a 60 min DNR or calcein-AM accumulation test could be used to detect MRP-mediated resistance. The MRP-specific modulator genistein could be used in combination with DNR, but not with calcein-AM. Vincristine (VCR) can be used to increase the cellular uptake of calcein-AM in MDR cells, but is not specific for MRP. Thus, although the combination of DNR with genistein appeared to be as sensitive as the combination of calcein-AM with VCR, the former may be used to probe specific MRP activity whereas the latter provides a combined (P-gp + MRP) functional MDR parameter. With these functional assays the role and relative importance of P-gp and MRP can be studied in, for example, haematological malignancies.

**Keywords**: P-glycoprotein; multidrug resistance-associated protein; daunorubicin; rhodamine; calcein; PSC833

Resistance to chemotherapy, whether it is intrinsic or acquired, is a major cause of failure in the curative treatment of haematological malignancies. Among the most active anti-cancer agents used in the treatment of haematological malignancies are some natural toxin-derived drugs, such as the anthracycline daunorubicin (DNR), the epipodophyllotoxin etoposide (VP-16) and the vinca alkaloid vincristine (VCR). Development of cross-resistance to these structurally and functionally unrelated drugs is called multidrug resistance (MDR). One type of MDR is caused by the overexpression of a plasma membrane protein, P-glycoprotein (P-gp), the product of the *MDR*1 gene (Endicott and Ling, 1989). P-gp functions as an ATP-dependent drug efflux pump, which results in lower intracellular drug concentrations and, hence, in drug resistance (Bradley *et al.*, 1988; Broxterman and Pinedo, 1991). In some haematological malignancies, such as acute myeloid leukaemias, non-Hodgkin's lymphomas and multiple myelomas, P-gp expression appears to be a poor prognostic factor which might predict refractoriness (Grogan *et al.*, 1993). *In vitro*, MDR can be circumvented by many relatively non-cytotoxic agents, such as calcium channel blockers and cyclosporins (Nooter *et al.*, 1990; Miller *et al.*, 1991). Therefore, the possibility of reversing MDR in the clinic has attracted considerable interest.

In addition to P-gp-mediated MDR, a number of *in vitro* drug-selected tumour cell lines which do not contain P-gp but nevertheless show the MDR phenotype have been described (Kuiper *et al.*, 1990; Versantvoort *et al.*, 1992). Several, but not all, of these non-P-gp MDR cell lines show overexpression of the gene encoding the multidrug resistance-associated protein (MRP), which was recently cloned by Cole *et al.* (1992) from the non-P-gp MDR small-cell lung cancer cell line H69AR. MRP is a 180–195 kDa membrane glycoprotein which, like P-gp, belongs to the ATP-binding cassette (ABC) superfamily of membrane transport proteins (Higgins, 1992). Gene transfection studies have demonstrated that MRP acts as a plasma membrane drug efflux pump and that *MRP* overexpression increases resistance to a wide spectrum of natural product drugs (Grant *et al.*, 1994; Zaman *et al.*, 1994; Broxterman and Versantvoort, 1995).

Detection and characterisation of resistant cells in clinical tumour samples could become an important diagnostic parameter in clinical practice. Detection of MDR is mostly performed by using gene-specific probes to detect mRNA (over)expression or monoclonal antibodies to detect the corresponding proteins (Fojo *et al.*, 1987; Van der Valk *et al.*, 1990). However, since MDR is caused by increased drug transport out of the cell, the best way of measuring would be to determine the free intracellular drug concentration. By measuring the drug accumulation in the cells, interpretation problems resulting from differences in post-translational modifications of the pump proteins (Center, 1985) and differences in passive drug uptake through the plasma membrane (Spoelstra *et al.*, 1992) can be avoided. An additional important advantage of a functional assay is that it can determine if the resistance can be modulated by resistance modifiers. In the case of modulation it may be considered to administer modifiers in conjunction with chemotherapy to the patient.

Flow cytometry is a readily applicable technique to assay MDR in leukaemias because several highly fluorescent molecules appear to be substrates for P-gp. The fluorescent probes rhodamine 123 (R123; Lampidis *et al.*, 1985), daunorubicin (DNR; van Acker *et al.*, 1993) and calcein acetoxymethyl ester (calcein-AM; Holló *et al.*, 1994) have been applied for P-gp detection using flow cytometry. To date, there are no reports in which the function of the novel MRP protein in leukaemias is assessed. Since *MRP* mRNA is expressed in leukaemias to a widely different extent (Burger *et al.*, 1994; Schuurhuis *et al.*, 1995), it will be important to study the functional effect of this protein in leukaemias. Therefore we investigated the specificity and sensitivity of fluorescent probes for detection of P-gp and MRP-mediated MDR. We compared different fluorescent probes by measuring the effect of modulators on their accumulation and retention in several drug-sensitive and P-gp- as well as MRP-mediated resistant cell lines using flow cytometry. Cell lines

Correspondence: HJ Broxterman, Department of Medical Oncology, BR 232, PO Box 7057, 1007 MB Amsterdam, The Netherlands

Received 10 January 1995; revised 10 April 1995; accepted 13 April 1995.

with a low resistance factor are included to test the sensitivity of the detection method, since it is likely that low resistance is important in clinical material (Schuurhuis *et al.*, 1995). In this report we present the results of experiments which compare daunorubicin, rhodamine and calcein-AM in P-gp and MRP MDR cells.

### Materials and methods

### Cell lines

The human epidermoid carcinoma cell line KB3-1 and its P-gp MDR sublines KB8 and KB8-5 [resistance factor (RF) for DNR about 2 and 5 respectively] were obtained from Dr I Roninson (KB3-1 and KB8) and from ATCC, Rockville, MD, USA (KB8-5). The non-small-cell lung cancer cell line SW-1573 and its non-P-gp MDR subline SW-1573/2R120 ($RF_{DNR} = 4$) have been described previously (Kuiper *et al.*, 1990). The S1(MRP) ($RF_{DNR} = 3.2$) subline was obtained by transfection of SW-1573 (S1) cells wtih an expression vector containing *MRP* cDNA and a neomycin resistance gene, followed by selection with geneticin (Zaman *et al.*, 1994). All the cell lines were cultured in Dulbecco's minimal essential medium (MEM) (Flow Labs, Irvine, UK) supplemented with 7.5% heat-inactivated fetal calf serum (FCS) (Gibco Europe, Paisley, UK). The human acute myelocytic leukaemia cell line HL60 and its MRP-overexpressing subline HL60/ADR ($RF_{DOX} = 80$) were obtained from Dr M Center (McGrath *et al.*, 1989). The human small-cell lung cancer cell line $GLC_4$ and its MRP-overexpressing sublines $GLC_4$-$ADR_2$ ($RF_{DNR} = 1.4$), $GLC_4$-$ADR_{pr}$ ($RF_{DNR} = 6.3$) and $GLC_4$-$ADR_{150}$ ($RF_{DNR} = 58$) have been characterised previously (Zijlstra *et al.*, 1987; Versantvoort *et al.*, 1995*a*). The $GLC_4$ and HL60 and their sublines were cultured in RPMI-1640 (Flow Labs) supplemented with 10% FCS. All the resistant cells were cultured in the presence of selecting drug until 2–10 days before the experiments were performed. The cell lines were mycoplasma free, as was regularly tested with the Mycoplasma TC kit (Gen-Probe, San Diego, CA, USA).

### Flow cytometry

Fluorescence was analysed with a FACScan flow cytometer (Becton Dickinson Medical Systems, Sharon, MA, USA), which was equipped with an argon laser. The fluorescence of 10 000 events was logarithmically measured at a laser excitation wavelength of 488 nm. The fluorescence of rhodamine 123, fluorescein isothiocyanate and calcein was collected through a 530 nm band-pass filter and fluorescence of daunorubicin was collected through a 575 nm filter. The logarithmically amplified signals were converted into values on a linear scale and expressed as relative fluorescence units (FU), from which the mean fluorescence was calculated.

### Drug accumulation and retention

Cells ($0.3-0.7 \times 10^6$) were incubated for 60 min at 37°C in 2 µM DNR (Sigma, St Louis, MO, USA) or 200 ng $ml^{-1}$ R123 (Sigma) or for 10 min in 0.5 µM calcein-AM (Molecular Probes, Eugene, OR, USA) with or without a modulator in medium A (growth medium without phenol red and bicarbonate buffer, but with 20 mM Hepes) + 10% FCS. PSC833 (Sandoz, Basle, Switzerland) at 2 µM, 200 µM genistein (Sigma) or 100 µM vincristine (Sigma) was used as a modulator. After the incubation the cells were washed in ice-cold medium A + 10% FCS. Non-specific binding of the drugs to the cells was measured by adding ice-cold drug-containing medium to the cells and washing them immediately.

In order to measure DNR and R123 retention, the cells were loaded with the drug for 60 min, and after washing the cells were incubated at 37°C in drug-free medium A + 10% FCS with or without the relevant modulator for 60 min. The efflux was stopped by centrifuging the cells and adding ice-cold medium.

### P-gp and MRP detection

In order to detect P-gp, viable cells were incubated for 1 h at room temperature with mouse monoclonal antibody MRK-16 (10 µg $ml^{-1}$) (kindly provided by Dr T Tsuruo) or a non-relevant mouse $IgG_{2a}$ (10 µg $ml^{-1}$) (Becton Dickinson). Cells were washed and for 30 min incubated in the dark with rabbit anti-mouse fluorescein isothiocyanate (1:100) (Dakopatts, Copenhagen, Denmark). In order to detect MRP, cells were permeabilised in 10% (v/v) Lysing solution G (Becton Dickinson) for 10 min and then incubated for 1 h at room temperature with rat monoclonal antibody MRPr1 (1.7 µg $ml^{-1}$) or a non-specific rat monoclonal antibody (provided by M Flens and G Zaman; Flens *et al.*, 1994). Antibody binding was detected with rabbit anti-rat fluorescein isothiocyanate (1:100) (Dakopatts). The mean of the fluorescence of the MRK16- or MRP-labelled cells was divided by the mean of the fluorescence of the non-relevant antibody-labelled cells.

### Flow-through system

The flow-through system has been described in detail previously (Lankelma *et al.*, 1990). Basically, a monolayer of approximately $10^7$ cells attached to a glass chamber (surface 50 $cm^2$, height 0.1 mm) was perfused with 2 µM DNR in medium A. The perfusion medium was passed over the cells at a constant flow of 200 µl $min^{-1}$ at 37°C until a steady-state level was reached. Then a series of pulse injections of modulators was introduced into the flowing perfusion medium, causing inhibition of the DNR efflux, which was measured at the outlet of the flow-through system as a decrease in the medium fluorescence of DNR. The fluorescence of DNR was measured by a fluorescence detector (type 821-FP, Jasco, Haschioji City, Japan) at excitation/emission wavelengths of 480 nm/560 nm.

### Results

### P-gp functional assays

Standard fluorescent probes for measuring the function of P-gp by flow cytometry are R123 and DNR (Lampidis *et al.*, 1985; Van Acker *et al.*, 1993). A more recently investigated probe for P-gp is calcein-AM (Holló *et al.*, 1994; Homolya *et al.*, 1993). In this study we compared these probes in order to assess their sensitivity and specificity for P-gp and MRP detection in accumulation and retention assays. First, we determined the accumulation ratio (the ratio of the fluorescence of drugs accumulated in the resistant and sensitive cell line) for these probes and the effect of the P-gp inhibitor PSC833 on their accumulation in the KB cell lines. Solubilisation of the cells with SDS after accumulation of the dyes confirmed that the effect of modulator on calcein and R123 fluorescence was indeed caused by an increased dye uptake. This excludes the possibility that the higher calcein or R123 fluorescence induced by the modulators is due to an altered intracellular drug distribution, resulting in a higher fluorescence of cells. In particular, the analysis of a slightly resistant cell line, such as KB8 (see last column of Table I for P-gp expression), is critical in these assays, since this low level of resistance appears to be relevant for leukaemias (Schuurhuis *et al.*, 1995). Table I shows that a reduced DNR accumulation can be detected in KB8 and KB8-5 cells compared with the sensitive parental KB3-1 cells. R123 and calcein accumulation were reduced in KB8-5, but not in the KB8 cell line. However, in the KB8 cell line a distinct effect of the P-gp modulator PSC833 on the R123 accumulation could be detected, and this effect of PSC833 was greater than the effect on DNR accumulation. In contrast, P-gp function in KB8 could not be detected by PSC833 modulation of calcein-AM uptake. Thus, the combination of PSC833 with

**Table I** Comparison of different fluorescent probes in P-gp MDR cells

| | *Accumulation ratio* | | | *Effect of PSC833* | | | |
|---|---|---|---|---|---|---|---|
| | *DNR* | *R123* | *Calcein* | *DNR* | *R123* | *Calcein* | *P-gp* |
| KB3-1 | 1 | 1 | 1 | 0.97 ± 0.04 | 0.97 ± 0.05 | 0.95 ± 0.07 | 1.0;1.0 |
| KB8 | 0.72 ± 0.19 | 1.04 ± 0.05 | 1.23 ± 0.19 | 1.12 ± 0.06[a] | 1.28 ± 0.14[a] | 0.97 ± 0.05 | 1.4;1.7 |
| KB8-5 | 0.27 ± 0.03[b] | 0.08 ± 0.01[b] | 0.19 ± 0.06[b] | 3.11 ± 1.18 | 11.98 ± 1.91[b] | 6.74 ± 1.68[b] | 12.8;12.3 |

Data are means ± s.d. of at least three independent experiments. Differences were tested for significance using a Student's *t*-test: [a]$P<0.05$; [b]$P<0.01$. The accumulation ratio is the basal fluorescence of resistant cells divided by the fluorescence of sensitive cells after drug accumulation. The effect of PSC833 (2 μM) is the ratio of drug accumulation with PSC833 divided by drug accumulation without PSC833. The amount of P-gp is calculated by dividing the mean of the fluorescence of the MRK16-labelled cells by the mean of the fluorescence of the non-relevant antibody-labelled cells (a ratio of 1.0 means that P-gp is undetectable).

**Table II** Comparison of R123 accumulation with retention in P-gp MDR cells

| *Cell lines* | *Effect of PSC833 on R123 accumulation* | *Effect of PSC833 on R123 retention* | |
|---|---|---|---|
| | | *Loaded with PSC* | *Loaded without PSC* |
| KB3-1 | 0.97 ± 0.05 | 1.02 ± 0.03 | 0.97–1.01 |
| KB8 | 1.28 ± 0.14[a] | 1.09 ± 0.11 | 1.47 ± 0.42 |
| KB8-5 | 11.98 ± 1.91[b] | 1.72 ; 1.97 | 11.96–15.23 |

Data are results of two independent experiments or are means ± s.d. of at least three independent experiments. Differences were tested for significance using a Student's *t*-test: [a]$P<0.05$; [b]$P<0.01$. Data are ratios of R123 fluorescence with PSC833 (2 μM) divided by R123 fluorescence without PSC833.

R123 appeared to be the most sensitive P-gp functional assay in these cell lines.

It has been suggested that the sensitivity of a functional assay can be enhanced by measuring drug retention instead of accumulation (Ross *et al.*, 1993). We therefore compared these different experimental set-ups with R123 in the KB cell lines. Figure 1 shows the time course of the R123 accumulation and retention with and without PSC833 in the KB8 cell line. The relative effect of PSC833 on the accumulation and retention was somewhat larger when the accumulation and efflux time respectively were longer (not shown). However, in order to compare the sensitivity of an accumulation and retention assay, time points of 60 min were chosen for practical reasons. Table II shows that no substantial increase in the sensitivity of the assays was obtained by measuring the effect of PSC833 on R123 retention, when cells were loaded without PSC833. However, when cells were loaded with PSC833 and subsequently effluxed, the effect of the modulator was even much smaller than in the accumulation assay in KB8-5 cells, and in KB8 cells it was hardly detectable. In contrast, when cells were loaded with 20 μM verapamil as modulator instead of 2 μM PSC833, no difference was observed between the effect of modulator on R123 accumulation and retention (data not shown).

In order to find an explanation for this apparent discrepancy between the effect of the clinically important modulator PSC833 on R123 accumulation and retention, KB8-5 cells were loaded with 2 μM DNR in a flow-through system until steady-state accumulation was reached. Then a pulse of 25 μM verapamil or 2 μM PSC833 was injected into the DNR-containing medium and the dynamic effect of cellular DNR influx by inhibition of P-gp (and DNR efflux after passage of the modulator) was recorded by measuring the fluorescence of DNR in the medium at the outlet of the flow-through system. DNR influx and efflux by cells were measured by the decrease and increase of the DNR fluorescence in the medium respectively (Figure 2). This experiment showed that the efflux of DNR is much more rapidly restored when verapamil instead of PSC833 is used as modulator. Apparently, PSC833 remains much longer in the cells than verapamil, which explains the slight modulator effects on the DNR efflux (see middle column of Table II). Therefore, PSC833 is not suitable for loading cells with drugs intended for subsequent drug efflux studies.

**Figure 1** Fluorescence of R123 after uptake and efflux in the KB8 cells. Cells were loaded with 200 ng ml$^{-1}$ R123 for 60 min with (– –●– –) and without (– –O– –) 2 μM PSC833. Retention of R123 was measured after suspending the cells in R123-free medium alone (—O—) or in the presence of 2 μM PSC833 (—●—).

**Figure 2** The effect of 2 μM PSC833 and 25 μM verapamil on the steady-state accumulation of 2 μM DNR in the KB8-5 cell line measured by the flow-through system (for further experimental detail see Lankelma *et al.*, 1990). The trace shows the decrease and increase in DNR fluorescence in the medium as a result of increased (influx) and decreased (efflux) cellular DNR uptake respectively.

### *Modulation of MRP-mediated transport*

The previous experiments showed that measuring the modulation of R123 accumulation by PSC833 is a convenient and sensitive functional assay for detecting P-gp activity. In order to establish whether PSC833 is also an efficient modulator for MRP, we first compared the effect of PSC833 with genistein, a compound which has been shown to inhibit

MRP- but not P-gp-mediated DNR efflux (Versantvoort et al., 1993). Table III shows that the effectiveness of PSC833 as a modulator of DNR accumulation varied among the different MRP-overexpressing cell lines. PSC833 was an effective modulator in the MRP-overexpressing HL60/ADR cells, but had no effect or only a slight effect on the accumulation of DNR in the other MRP-overexpressing cells. In conclusion, PSC833 appeared not to be strictly specific for P-gp, since in certain tumour cells it may also interact with DNR transport by MRP.

Since PSC833 was not a potent modulator in most MRP MDR cells, we investigated the use of genistein as a modulator in order to compare its effect on transport of DNR, R123 and calcein-AM. However, in preliminary experiments we found that genistein decreased the fluorescence of calcein as well as of R123 in sensitive cells. Because of this interaction, which was apparently unrelated to the presence of any drug efflux pump, genistein could not be used in combination with R123 or calcein-AM. Therefore, for R123 and calcein-AM we decided to use VCR as a modulator, since it has been shown previously that it modulates P-gp- as well as MRP-mediated drug efflux (Mülder et al., 1994).

### *R123 to probe MRP function*

The results of the R123 accumulation are summarised in Table IV. It appears that R123 is not a sensitive probe for detecting MRP-resistant cells, including the MRP transfectant. In fact, in a 60 min accumulation assay no consistently decreased R123 accumulation was observed. The GLC$_4$-ADR$_{150}$ and the HL60/ADR sublines accumulated even more R123 than the related parent cell lines. Nor was there an enhanced efflux of R123 in the GLC$_4$-ADR$_{150}$ compared with the GLC$_4$ parent cell line, as illustrated in Figure 3. Only in the SW-1573/2R120 and the S1(MRP) sublines could a small R123 accumulation defect be detected. Since PSC833 was an effective modulator of DNR transport in the MRP-overexpressing HL60/ADR cell line, as shown before (Table III), we tested the combination of R123 and PSC833 in these cells. However, PSC833 had only a slight effect on R123 accumulation in this cell line. We also tested VCR as a modulator of R123 accumulation in the transfectant S1(MRP) and in the sensitive cell line SW-1573, but again a decrease in the fluorescence of R123 was seen in both cell lines (data not shown), which suggests a non-specific interaction among these drugs, unrelated to the function of the MRP efflux pump. In conclusion, R123 appeared to be an

**Figure 3** Uptake and retention of R123 in the GLC$_4$ and GLC$_4$-ADR$_{150}$ cells. GLC$_4$ (- -■- -) and GLC$_4$-ADR$_{150}$ (- -▲- -) cells were loaded for 60 min with 200 ng ml$^{-1}$ R123. Retention of R123 was measured after suspending the GLC$_4$ (—■—) and GLC$_4$-ADR$_{150}$ (—▲—) cells in R123-free medium.

**Table III** Comparison of the effect of PSC833 and genistein on DNR accumulation in MRP-overexpressing cells

| | *PSC833* | *Genistein* |
|---|---|---|
| SW-1573 | 1.00 ± 0.09 | 0.99 ± 0.01 |
| S1(MRP) | 1.05 ± 0.04 | 1.19 ± 0.07$^a$ |
| SW-1573 2R120 | 1.13 ± 0.06 | 1.21 ± 0.06$^b$ |
| GLC$_4$ | 1.02 ± 0.02 | 1.01 ± 0.06 |
| GLC$_4$-ADR$_{150}$ | 1.05 ± 0.05 | 2.24 ± 0.36$^a$ |
| HL60 | 1.04 ± 0.06 | 1.05 ± 0.05 |
| HL60 ADR | 1.53 ± 0.06$^b$ | 1.61 ± 0.25$^a$ |

Data are ratios of DNR accumulation with a modulator divided by DNR accumulation without a modulator. Data are means ± s.d. of at least three independent experiments. Differences were tested for significance using a Student's $t$-test: $^aP<0.05$; $^bP<0.01$.

**Table V** MRP overexpression and the effect of genistein on DNR accumulation and retention in the GLC$_4$ and resistant sublines

| | *Effect of genistein* | | *Accumulation* | *MRP* |
|---|---|---|---|---|
| | *Accumulation* | *Retention* | *ratio* | *expression* |
| GLC$_4$ | 1.01 ± 0.08 | 1.12 ± 0.04$^a$ | 1 | 1.5:1.6 |
| GLC$_4$-ADR$_2$ | 1.09 ± 0.07 | 1.19 ± 0.04$^a$ | 0.88 ± 0.16 | 1.8:2.0 |
| GLC$_4$-ADR$_{pr}$ | 1.35–1.47 | 1.28–1.36 | 0.53 ± 0.14 | 2.1:2.5 |
| GLC$_4$-ADR$_{150}$ | 2.24 ± 0.36$^a$ | 2.08 ± 0.31$^a$ | 0.13 ± 0.06$^b$ | 6.6:8.2 |

Data are means ± s.d. of three independent experiments. For accumulation studies cells were accumulated with DNR (2 μM) for 60 min with and without genistein. Differences were tested for significance with Student's $t$-test: $^aP<0.05$; $^bP<0.01$. For retention studies the cells were loaded with DNR for 60 min and then cells were incubated for another 60 min in DNR-free medium with and without genistein. The effect of genistein is the ratio of the DNR fluorescence with genistein and the fluorescence without genistein. The accumulation ratio is the basal fluorescence of resistant cells divided by the fluorescence of sensitive cells after drug accumulation. MRP expression is calculated by dividing the mean of the fluorescence of the MRPr1-labelled cells by the mean of the fluorescence of the non-relevant antibody-labelled cells (a ratio of 1.0 means that MRP is undetectable).

**Table IV** Comparison of fluorescent probes in MRP-overexpressing cells

| | *Accumulation ratio* | | | *Effect of modulator* | | *MRP* |
|---|---|---|---|---|---|---|
| | *DNR* | *Calcein* | *R123* | *DNR* | *Calcein* | *expression* |
| SW-1573 | 1 | 1 | 1 | 0.99 ± 0.01 | 1.00 ± 0.13 | 1.8:2.2 |
| S1(MRP) | 0.56 ± 0.20 | 0.44 ± 0.03$^b$ | 0.85 ± 0.12 | 1.19 ± 0.07$^a$ | 1.42 ± 0.14$^a$ | 3.2:3.1 |
| SW-1573 2R120 | 0.42 ± 0.04$^b$ | 0.47 ± 0.04$^b$ | 0.93 | 1.21 ± 0.06$^b$ | 1.29 ± 0.03$^b$ | 3.1:2.5 |
| GLC$_4$ | 1 | 1 | 1 | 1.01 ± 0.08 | 0.86 ± 0.09 | 1.5:1.5 |
| GLC$_4$-ADR$_{150}$ | 0.13 ± 0.06$^b$ | 0.15 ± 0.14$^b$ | 1.72 ± 0.14$^a$ | 2.24 ± 0.36$^a$ | 2.36 ± 0.67$^a$ | 6.6:8.2 |
| HL60 | 1 | 1 | 1 | 1.05 ± 0.05 | 0.90 ± 0.06 | 2.1:2.5 |
| HL60 ADR | 0.34–0.49 | 0.17 ± 0.05$^b$ | 1.65 | 1.61 ± 0.25$^a$ | 4.46 ± 0.63$^a$ | 8.2:7.4 |

Data are means ± s.d. of at least three independent experiments. Differences were tested for significance with Student's $t$-test: $^aP<0.05$; $^bP<0.01$. The accumulation ratio is the basal fluorescence of resistant cells divided by the fluorescence of sensitive cells after drug accumulation. Genistein (200 μM) was used as a modulator for DNR accumulation and VCR (100 μM) was used as a modulator for the calcein accumulation. MRP expression is calculated by dividing the mean of the fluorescence of the MRPr1-labelled cells by the mean of the fluorescence of the non-relevant antibody-labelled cells (a ratio of 1.0 means that MRP is undetectable). For the SW-1573 series the negative control was obtained by incubation without a primary antibody.

insensitive probe for detecting MRP function. Therefore, we only tested the modulator effects systematically on DNR and calcein accumulation.

*DNR and calcein to probe MRP function*

In contrast to R123, DNR and calcein-AM could be used to detect MRP-mediated resistance. DNR and calcein-AM appeared to have similar accumulation defects in all the MRP-overexpressing cell lines, except in the HL60/ADR subline (Table IV). In these cells the accumulation defect of calcein and the effect of the modulator on calcein accumulation were greater than the effect on DNR. Since genistein is a specific modulator of MRP-mediated transport and cannot be used together with calcein (see above), the preferred combination for specifically detecting the MRP function is DNR and genistein.

*Sensitivity of MRP detection*

In order to analyse the sensitivity of the preferred combination of DNR and genistein for detecting the MRP function, DNR accumulation and retention were tested in the $GLC_4$ series of cell lines with increasing MRP expression. Table V shows that DNR accumulation was progressively decreased in the cell lines with increasing MRP expression. Also, the effect of genistein on DNR accumulation and retention was greater in cells with increasing resistance and MRP expression. However, the retention assay was not more sensitive than the accumulation assay. Nor did the method of loading the cells before efflux, with or without genistein, alter the effect of genistein on DNR retention (data not shown). From these data it can be inferred that this assay was not sensitive enough to distinguish between $GLC_4$ and $GLC_4$-$ADR_2$ cells, which are 1.4-fold resistant to DNR (Versantvoort *et al.*, 1995*a*). The $GLC_4$-$ADR_{pr}$ and $GLC_4$-$ADR_{150}$ sublines which are 6.3- and 58-fold resistant to daunorubicin respectively, could easily be detected. Notably in the 3-fold resistant S1(MRP) cell line, MRP-related drug resistance could be detected in the DNR accumulation assay, as shown in Table IV.

### Discussion

Many fluorescent probes (DNR, DOX, R123, Hoechst 33342, BCECF-AM, calcein-AM, FURA-2-AM, etc.) are available to detect P-gp-mediated MDR. These probes have been used to detect P-gp by flow cytometry and laser scan microscopy (Schuurhuis *et al.*, 1991; Homolya *et al.*, 1993; Van Acker *et al.*, 1993), and the function of P-gp has been characterised in many human tumour cell lines. However, until now systematic studies into the sensitivity and specificity of these probes for detecting P-gp activity have not yet been reported. Moreover, this issue has become more complicated since it is now recognised that at least one other drug pump, namely MRP, may be involved in MDR. Both P-gp- and MRP-overexpressions appear to be important resistance mechanisms in haematological malignancies (Burger *et al.*, 1994; Kuss *et al.*, 1994; Schuurhuis *et al.*, 1995).

In order to gain more insight into the specificity and sensitivity of methods of detecting MDR by flow cytometry, we studied three fluorescent probes, R123, DNR and calcein-AM, combined with three different modulators, PSC833, genistein and VCR, in P-gp- and MRP-overexpressing cells. Each of these combinations has its own merits and drawbacks, as discussed below. The fluorescent probes were selected for the following reasons. DNR is a cytotoxic drug with favourable fluorescent properties used in the treatment of acute myeloid leukaemia (AML) (Schuurhuis *et al.*, 1995). Calcein-AM has been reported to be a good probe for P-gp because it is a hydrophobic non-fluorescent molecule which rapidly permeates the plasma membrane of cells. By cleavage of the ester bonds by intracellular esterases it is transformed to calcein, which is a highly fluorescent, non-membrane-permeable free acid. Moreover, since calcein, in contrast to calcein-AM, is not transported by P-gp, it is well retained by cells, with a fluorescence which is essentially insensitive to changes in pH, as well as to changes in $Ca^{2+}$ or $Mg^{2+}$ concentration (Homolya *et al.*, 1993). R123 is also a sensitive probe for P-gp, but the accumulation of R123 will be dependent on pH and mitochondrial storage capacity. R123 is not well retained by cells and some leakage of the probe occurs upon the transfer of accumulated cells into dye-free medium at 0°C (Holló *et al.*, 1994).

In this study we compared these different fluorescent dyes by measuring the effect of modulators on their accumulation in P-gp as well as MRP MDR cells. PSC833, a cyclosporin analogue, was chosen as a modulator for P-gp because it has acceptable toxicity when given to patients in concentrations known to reverse P-gp-mediated drug resistance *in vitro*. Interestingly, this study provides evidence that the action of PSC833 on P-gp-mediated DNR pumping is much longer lasting than the action of verapamil (Figure 2), presumably because PSC833 remains longer at the P-gp binding site in the cells. This might also have relevance for clinical modulation studies. Genistein was chosen as a modulator to detect MRP function in cells because it has been shown to inhibit specifically MRP-mediated DNR transport. However, genistein in the presently used concentration is too toxic for clinical applications (Versantvoort *et al.*, 1993).

We first found that the R123/PSC833 combination provided the most sensitive test for detecting P-gp-mediated MDR. This P-gp functional assay is very sensitive since the slightly resistant KB8 cell line ($RF_{DNR} \approx 2$) could be detected by PSC833 modulation. This is despite the fact that the KB8 cell line had no accumulation defect for R123. One possible explanation is that the number of mitochondria is somewhat increased in this cell line. Remarkably, no modulation of calcein accumulation could be detected in the KB8 cell line, whereas in the KB8-5 cell line calcein appeared to be a more sensitive probe than DNR. The reason for this discrepancy is not known. For the time being, when clinical samples are being tested for P-gp function, we would recommend that R123 be used in combination with 2 $\mu$M PSC833.

Next, we tested these probes in MRP-mediated drug-resistant cell lines. A remarkable result was that R123, the most sensitive probe for P-gp, was not a suitable probe for sensitive detection of MRP-mediated drug resistance in cell lines. Although MRP-overexpressing cells are resistant to R123 (Zaman *et al.*, 1994) and the highly MRP-overexpressing COR-L23 R cell line showed increased efflux of R123 (Twentyman *et al.*, 1994), no accumulation defect was found in the resistant cell lines $GLC_4$-$ADR_{150}$ and HL60/ADR after 60 min incubation with R123. It might be surprising that we found that R123 accumulation after 60 min is higher in some MRP-overexpressing cell lines than in the parental sensitive cell lines. This may be due to R123 being trapped in cytosolic vesicles containing MRP, as suggested by Cole *et al.* (1992). In addition, in contrast to P-gp, an accumulation defect for R123 can only be found after a longer incubation time (Twentyman *et al.*, 1994). Since there is evidence that the function of drug pumps in clinical samples, such as in AML cells, may deteriorate after storage, we prefer short-term (60 min) drug accumulation assays. Under these conditions R123 cannot be used as a probe for detecting MRP-mediated resistance and therefore R123 can be regarded as P-gp specific.

Calcein-AM appeared to be an effective probe for MRP-mediated resistance but the specific MRP modulator genistein decreased calcein fluorescence in sensitive cells and could not be used as a modulator. For this reason VCR, a modulator of P-gp- and MRP-mediated resistance (Mülder *et al.*, 1994), was used to modulate the calcein accumulation in MRP-resistant cells. A disadvantage of the combination of calcein-AM and VCR is that MRP-related resistance cannot be distinguished from P-gp-related resistance. Since the combination of DNR and genistein appeared to be as sensitive as the combination of calcein-AM and VCR, and since DNR modulation by genistein is specific for MRP, we prefer the combination of DNR and genistein for testing specific MRP-mediated resistance.

Some data suggested a different spectrum of MRP activity in the human leukaemia HL60 ADR cells since, in contrast to the other MRP-overexpressing cells, PSC833 is a potent modulator of the DNR accumulation, and it also appeared that calcein-AM is a more effective probe than DNR when this cell line is compared with the other MRP MDR cell lines. For this reason, calcein-AM with VCR as modulator may yet be a very useful alternative for testing leukaemias.

Recent studies have suggested that anti-cancer drugs may be conjugated in cells and that these conjugates may be the substrate for the MRP transporter (Jedlitschky *et al.*, 1994). Resistance would then depend on the conjugating enzymes as well as on MRP expression. However, it is important to note here that all the MRP-overexpressing cells tested had an accumulation defect for calcein. It has been reported that P-gp extrudes calcein-AM directly from the plasma cell membrane without entering the cellular cytoplasm and that calcein is not a probe for P-gp (Homolya *et al.*, 1993; Holló *et al.*, 1994). If MRP extrudes calcein-AM by a similar mechanism to P-gp, then our data would suggest that MRP may extrude hydrophobic drugs in a non-conjugated form from the plasma membrane compartment. Elucidation of the mechanism of extrusion by MRP of different classes of drugs needs more research (Versantvoort *et al.*, 1995*b*).

In the $GLC_4$ series the degree of MRP expression was in accordance with drug resistance (Versantvoort *et al.*, 1995*a*) as well as with the DNR accumulation defect. However, the SW-1573 sensitive cell line had a relatively high level of MRP, as detected with the MRPr1 antibody, but genistein had no effect on DNR accumulation. One possible explanation for this apparent discrepancy is that cells had to be permeabilised before being stained with MRPr1, since MRPr1 is a monoclonal antibody which recognises an internal epitope (Flens *et al.*, 1994). Therefore, no distinction could be made between functional MRP in the plasma cell membrane and MRP in structures such as the Golgi system (Flens *et al.*, 1994). Since MRP staining of the plasma membrane SW-1573 cells was undetectable by immunocytochemistry (Flens *et al.*, 1994), it could well be that this cell line contains more non-functional MRP than other sensitive cell lines. In future, monoclonal antibodies directed against outer epitopes of MRP will clarify this point. Alternatively, a role of intracellular MRP might be inferred from data on the *MRP*-overexpressing cell line H69 AR, which showed an altered intracellular distribution of doxorubicin but no doxorubicin accumulation defect (Cole *et al.*, 1992). For this reason we also explore another approach to detect P-gp- as well as MRP-mediated MDR, by using laser scan microscopy to detect the effect of modulators on the amount of nuclear anthracycline fluorescence in relation to P-gp and MRP expression (Broxterman *et al.*, 1994; Schuurhuis *et al.*, 1991). Preliminary data from these studies show that in AML P-gp- as well as MRP-mediated MDR is at the level of KB8 resistance (Schuurhuis *et al.*, 1995).

In conclusion, the present study shows that R123 accumulation in combination with PSC833 is a specific and sensitive test for detecting low levels of P-gp-mediated resistance, which could be of importance in clinical practice. For detection of MRP-mediated resistance, R123 did not appear to be a sensitive probe in a 60 min accumulation assay, whereas the combination of DNR with genistein was more sensitive and selective. The use of calcein-AM with an appropriate modulator (VCR) may be an alternative. The finding that the P-gp-inhibitory action of PSC833 lasts longer than that of verapamil may pertain to the use of PSC833 for modulating MDR in clinical studies.

**Acknowledgements**
This study was supported by grants from the Dutch Cancer Foundation (IKA-VU-93-626) and the VSB grant programme.

**References**

BRADLEY G, JURANKA PF AND LING V. (1988). Mechanism of multidrug resistance. *Biochim. Biophys. Acta*, **948**, 87–128.

BROXTERMAN HJ AND PINEDO HM. (1991). Energy metabolism in multidrug resistant tumor cells: a review. *J. Cell Pharmacol.*, **2**, 239–247.

BROXTERMAN HJ AND VERSANTVOORT CHM. (1995). Pharmacology of drug transport in multidrug resistant tumor cells. In *Alternative Mechanisms of Multidrug Resistance in Cancer*, Kellen JA (ed.), pp. 67–80. Birkhäuser: Boston.

BROXTERMAN HJ, KUIPER CM, EEKMAN CA, FELLER N, SCHUURHUIS GJ, PINEDO HM, BAAK JPA AND LANKELMA J. (1994). Functional detection of multidrug resistant tumour cells using flow cytometry (FC) and laser scan microscopy (LSM). *Anal. Cell. Pathol.*, **6**, 214.

BURGER H, NOOTER K, ZAMAN GJR, SONNEVELD P, VAN WINGERDEN KE, OOSTRUM RG AND STOTER G. (1994). Expression of the multidrug resistance protein (*MRP*) in acute and chronic leukemias. *Leukemia*, **6**, 990–997.

CENTER MS. (1985). Mechanisms regulating cell resistance to adriamycin. Evidence that drug accumulation in resistant cells is modulated by phosphorylation of a plasma membrane protein. *Biochem. Pharmacol.*, **34**, 1471–1476.

COLE SPC, BHARDWAJ G, GERLACH JH, MACKIE JE, GRANT CE, ALMQUIST KC, STEWART AJ, KURZ EU, DUNCAN AMV AND DEELEY RG. (1992). Overexpression of a novel transporter gene in a multidrug resistant human lung cancer cell line. *Science*, **258**, 1650–1654.

ENDICOTT JA AND LING V. (1989). The biochemistry of P-glycoprotein-mediated multidrug resistance. *Annu. Rev. Biochem.*, **58**, 137–171.

FLENS MJ, IZQUIERDO MA, SCHEFFER GL, FRITZ JM, MEIJER CJLM, SCHEPER RJ AND ZAMAN GJR. (1994). Immunochemical detection of the multidrug resistance-associated protein MRP in human multidrug-resistant tumor cells by monoclonal antibodies. *Cancer Res.*, **54**, 4557–4563.

FOJO A, UEDA K, SLAMON DJ, POPLACK DG, GOTTESMAN MM AND PASTAN I. (1987). Expression of a multidrug resistant gene in human tumors and tissues. *Proc. Natl Acad. Sci. USA*, **84**, 265–269.

GRANT CE, VALDIMARSSON G, HIPFNER DR, ALMQUIST KC, COLE SPC AND DEELEY RG. (1994). Overexpression of multidrug resistance-associated protein (MRP) increases resistance to natural product drugs. *Cancer Res.*, **54**, 357–361.

GROGAN TM, SPIER CM, SALMON SE, MATZNER M, RYBSKI J, WEINSTEIN RS, SCHEPER RJ AND DALTON WS. (1993). P-glycoprotein expression in human plasma cell myeloma: correlation with prior chemotherapy. *Blood*, **81**, 490–495.

HIGGINS CF. (1992). ABC transporters from microorganisms to man. *Annu. Rev. Cell Biol.*, **8**, 67–113.

HOLLÓ Z, HOMOLYA L, DAVIS CM AND SARKADI B. (1994). Calcein accumulation as a fluorometric functional assay of the multidrug transporter. *Biochim. Biophys. Acta*, **1191**, 384–388.

HOMOLYA L, HOLLÓ Z, GERMANN UA, PASTAN I, GOTTESMAN MM AND SARKADI B. (1993). Fluorescent cellular indicators are extruded by the multidrug resistance protein. *J. Biol. Chem.*, **29**, 21493–21496.

JEDLITSCHKY G, LEIER I, BUCHHOLZ U, CENTER M AND KEPPLER D. (1994). ATP-dependent transport of *S*-conjugates by the multidrug resistance-associated protein (MRP). *Cancer Res.*, **54**, 4833–4836.

KUIPER CM, BROXTERMAN HJ, BAAS F, SCHUURHUIS GJ, HAISMA HJ, SCHEFFER GL, LANKELMA J AND PINEDO HM. (1990). Drug transport variants without P-glycoprotein overexpression from a human squamous lung cancer cell line after selection with doxorubicin. *J. Cell. Pharmacol.*, **1**, 35–41.

KUSS BJ, DEELEY RC, COLE SPC, WILLMAN CL, KOPECKY KJ, WOLMAN SR, EYRE HJ, LANE SA, NANCARROW JK, WITHMORE SA AND CALLEN DF. (1994). Deletion of the gene for multidrug resistance in acute myeloid leukaemia with inversion in chromosome 16: prognostic implications. *Lancet*, **343**, 1531–1534.

LAMPIDIS TJ, MUNCK JN, KRISHAN A AND TAPIERO H. (1985). Reversal of resistance to rhodamine in adriamycin-resistant Friend leukemia cells. *Cancer Res.*, **45**, 2626–2631.

LANKELMA J, SPOELSTRA EC, DEKKER H AND BROXTERMAN HJ. (1990). Evidence for daunorubicin efflux from multidrug-resistant $2780^{AD}$ human ovarium carcinoma cells against a concentration gradient. *Biochim. Biophys. Acta*, **1055**, 217–222.

McGRATH T. LATOUD C. ARNOLD ST. SAFA AR. FELSTED RL AND CENTER MS. (1989). Mechanisms of multidrug resistance in HL60 cells. Analysis of resistance associated proteins and levels of *mdr* gene expression. *Biochem. Pharmacol.*, **38**, 3611-3619.

MILLER TP. GROGAN TM. DALTON WS. SPIER CM. SCHEPER RJ AND SALMON SE. (1991). P-glycoprotein expression in malignant lymphoma and reversal of clinical drug resistance with chemotherapy plus high dose verapamil. *J. Clin. Oncol.*, **9**, 17-24.

MÜLDER HS. LANKELMA J. DEKKER H. BROXTERMAN HJ AND PINEDO HM. (1994). Daunorubicin efflux against a concentration gradient in non-P-glycoprotein multidrug resistance lung-cancer cells. *Int. J. Cancer*, **59**, 275-281.

NOOTER K. SONNEVELD P. OOSTRUM R. HERWEIJER H. HAGENBEEK T AND VALERIO D. (1990). Overexpression of the MDR1 gene in blast cells with acute myeloid leukemia is associated with decreased anthracycline accumulation that can be restored by cyclosporin A. *Int. J. Cancer*, **45**, 263-268.

ROSS DD. WOOTEN PJ. SRIDHARA R. ORDONEZ JV. LEE EJ AND SCHIFFER SA. (1993). Enhancement of daunorubicin accumulation, retention and cytotoxicity by verapamil or cyclosporin A in blast cells from patients with previously untreated acute myeloid leukemia. *Blood*, **82**, 1288-1299.

SCHUURHUIS GJ. BROXTERMAN HJ. DE LANGE JHM. PINEDO HM. VAN HEIJNINGEN THM. KUIPER CM. SCHEFFER GL. VAN KALKEN CK. BAAK JPA AND LANKELMA J. (1991). Early multidrug resistance, defined by changes in intracellular doxorubicin distribution, independent of P-glycoprotein. *Br. J. Cancer*, **64**, 857-861.

SCHUURHUIS GJ. BROXTERMAN HJ. OSSENKOPPELE GJ. BAAK JPA. EEKMAN CA. KUIPER CM. FELLER N. VAN HEIJNINGEN THM. KLUMPER E. PIETERS R. LANKELMA J AND PINEDO HM. (1995). Functional multidrug resistance phenotype associated with combined overexpression of Pgp/*MDR*1 and *MRP* together with cytosine-arabinoside sensitivity may predict clinical response in acute myeloid leukemia. *Clin. Cancer Res.*, **1**, 81-93.

SPOELSTRA EC. WESTERHOFF HV. DEKKER H AND LANKELMA J. (1992). Kinetics of daunorubicin transport by P-glycoprotein of intact cancer cells. *Eur. J. Biochem.*, **207**, 567-579.

TWENTYMAN PR. RHODES T AND RAYNER S. (1994). A comparison of rhodamine 123 accumulation and efflux in cells with P-glycoprotein-mediated and MRP-associated multidrug resistance phenotypes. *Eur. J. Cancer*, **9**, 1360-1364.

VAN ACKER KL. VAN HOVE LM AND BOOGAERTS MA. (1993). Evaluation of flow cytometry for multidrug resistance detection in low resistance K562 cells using daunorubicin and monoclonal antibodies. *Cytometry*, **14**, 736-746.

VAN DER VALK P. VAN KALKEN CK. KETELAARS H. BROXTERMAN HJ. SCHEFFER GL. KUIPER CM. TSURUO T. LANKELMA J. MEIJER CJLM. PINEDO HM AND SCHEPER RJ. (1990). Distribution of multidrug resistance-associated P-glycoprotein in normal and neoplastic human tissues. *Ann. Oncol.*, **1**, 56-64.

VERSANTVOORT CHM. BROXTERMAN HJ. PINEDO HM. DE VRIES EGE. FELLER N. KUIPER CM AND LANKELMA J. (1992). Energy-dependent processes involved in reduced drug accumulation in multidrug resistant human lung cancer cell lines without P-glycoprotein expression. *Cancer Res.*, **52**, 17-23.

VERSANTVOORT CHM. SCHUURHUIS GJ. PINEDO HM. EEKMAN CA. KUIPER CM. LANKELMA J AND BROXTERMAN HJ. (1993). Genistein modulates the decreased drug accumulation in non-P-glycoprotein mediated multidrug resistant tumour cells. *Br. J. Cancer*, **68**, 939-946.

VERSANTVOORT CHM. WITHOFF S. BROXTERMAN HJ. KUIPER CM. SCHEPER RJ. MULDER NH AND DE VRIES EGE. (1995*a*). Resistance associated factors in human small cell lung carcinoma $GLC_4$ sublines with increasing adriamycin resistance. *Int. J. Cancer*, **61**, 375-380.

VERSANTVOORT CHM. BROXTERMAN HJ. BAGRIJ T. SCHEPER RJ AND TWENTYMAN P. (1995*b*). Regulation by glutathione of drug transport in multidrug resistant human lung cancer cell lines overexpressing MRP. *Br. J. Cancer*, **72** (in press).

ZAMAN GJR. FLENS MJ. VAN LEUSDEN MR. DE HAAS M. MÜLDER HS. LANKELMA J. PINEDO HM. SCHEPER RJ. BAAS F. BROXTERMAN HJ AND BORST P. (1994). The human multidrug resistance-associated protein MRP is a plasma membrane drug-efflux pump. *Proc. Natl Acad. Sci. USA*, **91**, 8822-8826.

ZIJLSTRA JG. DE VRIES EGE AND MULDER NH. (1987). Multifactorial drug resistance in an Adriamycin-resistant human small cell lung carcinoma cell line. *Cancer Res.*, **47**, 1780-1784.