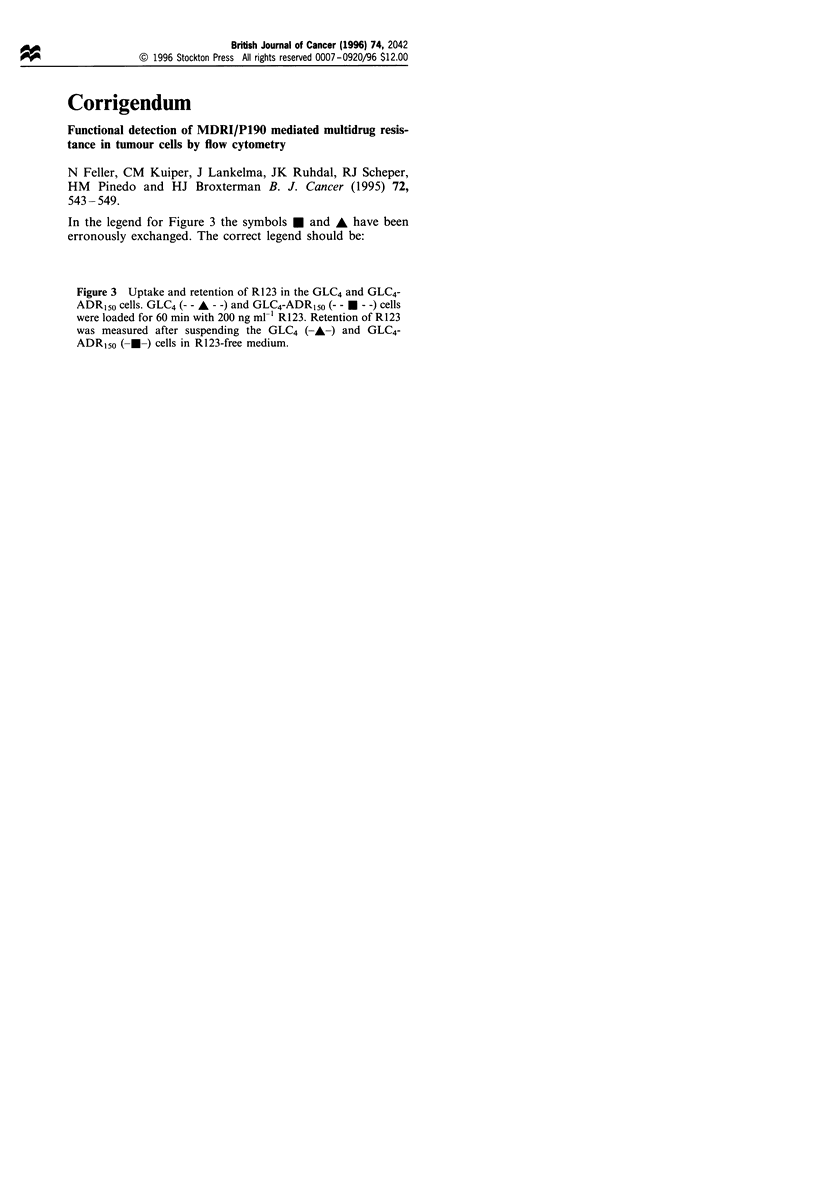# Functional detection of MDRI/P190 mediated multidrug resistance in tumour cells by flow cytometry

**Published:** 1996-12

**Authors:** 


British Journal of Cancer (1996) 74, 2042
© 1996 Stockton Press All rights reserved 0007-0920/96 $12.00

### Corrigendum

**Functional detection of MDRI/P190 mediated multidrug resistance in tumour cells by flow cytometry**

N Feller, CM Kuiper, J Lankelma, JK Ruhdal, RJ Scheper, HM Pinedo and HJ Broxterman *B. J. Cancer* (1995) **72,** 543-549.

In the legend for Figure 3 the symbols ■ and ▲ have been erronously exchanged. The correct legend should be:

**Figure 3** Uptake and retention of R123 in the $GLC_4$ and $GLC_4$-$ADR_{150}$ cells. $GLC_4$ (- - ▲ - -) and $GLC_4$-$ADR_{150}$ (- - ■ - -) cells were loaded for 60 min with 200 ng $ml^{-1}$ R123. Retention of R123 was measured after suspending the $GLC_4$ (-▲-) and $GLC_4$-$ADR_{150}$ (-■-) cells in R123-free medium.